# Primary liposarcoma of the pericardium: report of a rare case with clinicopathological features and imaging insights

**DOI:** 10.1186/s12872-026-05809-6

**Published:** 2026-04-29

**Authors:** Juan David Rojas-Perdomo, Santiago Uribe-Díaz, Manuel García-Bustos, Esaú Álvarez-Lora, Carlos Fuentes-Pérez, Martha Velasco-Morales, Wilmer Cely-Cely, Jorge Luis Rodríguez-Sarmiento, Henry Robayo-Amortegui, Fernán Mendoza-Beltrán

**Affiliations:** 1 Department of Cardiology, Fundación Clínica Shaio, Dg 115ª # 70c-75, Bogotá, D.C Colombia; 2 Department of Internal Medicine, Universidad Nacional, Bogotá, Colombia; 3 Internal Medicine, Universidad de Cartagena, Cartagena, Colombia; 4 Radiology Department, Fundación Clínica Shaio, Bogotá, Colombia; 5 School of medicine, Universidad de La Sabana, Chía, Cundinamarca Colombia; 6 Department of Critical Care Medicine, Extracorporeal Life Support Unit, Fundación Clínica Shaio, Bogotá , D.C Colombia; 7 Clinical and Pathology Laboratory, Keralty Group, Clinica Colsanitas, Bogotá, Colombia

**Keywords:** Primary cardiac tumor, Liposarcoma, Echocardiogram, Cardiac MRI

## Abstract

**Background:**

Primary cardiac tumors are extremely uncommon, and malignant variants are even rarer. Among them, pericardial liposarcoma is exceptionally infrequent, with very few cases reported worldwide. Because of its nonspecific clinical presentation and the complexity of cardiac anatomy, diagnosis is often delayed. This case is notable due to the tumor’s unusual pericardial origin and demonstrates the critical role of multimodal cardiac imaging in identifying rare primary cardiac malignancies.

**Case presentation:**

A 56-year-old woman with a history of arterial hypertension and prior SARS-CoV-2 infection presented with progressive dyspnea and chest pain of several weeks’ duration. Physical examination and initial laboratory tests were nonspecific. Transthoracic echocardiography revealed a large heterogeneous pericardial mass. Further evaluation with computed tomography and cardiac magnetic resonance imaging demonstrated a lesion with mixed solid and fatty components, closely associated with the pericardium. Surgical biopsy with histopathological and immunohistochemical analysis confirmed the diagnosis of undifferentiated liposarcoma of probable pericardial origin. The patient was referred for specialized oncologic management; however, her clinical course was unfavorable, and she ultimately experienced disease progression.

**Conclusions:**

This case illustrates the diagnostic challenges associated with rare primary cardiac malignancies and emphasizes the importance of integrated multimodal imaging in characterizing pericardial tumors. Increased clinical awareness and further research are needed to optimize therapeutic strategies and improve outcomes in patients with pericardial liposarcoma.

## Introduction

Cardiac tumors are rare findings, with prevalences in surgical series ranging up to 0.73% [1]. They can be classified benign and malignant tumors, with the latter further categorized as primary or secondary. When the findings are primary tumors, the prevalence is as low as 0.02% according to autopsy series [2]. To the authors’ knowledge, there are less than 20 cases of pericardial liposarcoma reported in indexed journals. We present a case of primary liposarcoma of the pericardium, an unusual pathology with an unfortunate course [1, 3].

## Case report

We present the case of a 56-year-old female with a history of arterial hypertension and a recent SARS-CoV-2/COVID-19 infection, managed on an outpatient basis approximately four months before her recent visit to the emergency department (ED), without known sequelae. Upon admission to the emergency department, she reported four weeks of dry cough, progressively worsening exertional dyspnea, paroxysmal nocturnal dyspnea, and intermittent chest pain. On admission, vital signs were as follows: heart rate 65 beats per minute (bpm), respiratory rate 20 respirations per minute (rpm), blood pressure 96/72 mmHg, oxygen saturation 96% in ambient air, temperature 36.2 °C. Physical examination revealed jugular venous distention, muffled heart sounds, and Grade I lower extremity edema with pitting.

**Fig. 1:**
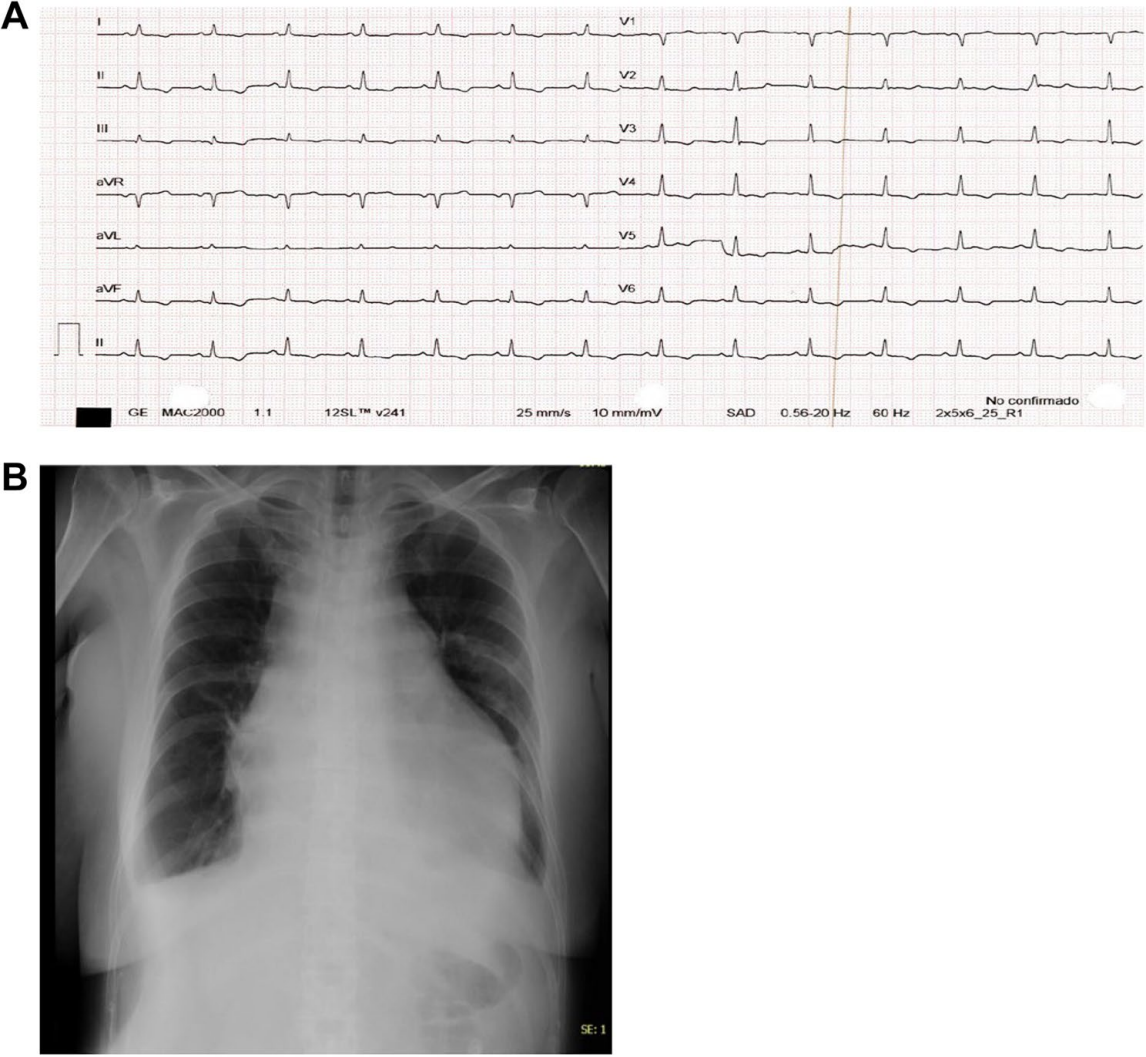
Admission electrocardiogram and chest X-ray. **A** 12-lead ECG demonstrating sinus rhythm (70 bpm) with generalized low QRS voltage andelectrical alternans in V2-V3, indicative of a large pericardial effusion. This corresponds to the electrocardiogram. **B** Frontal chest X-ray showing a significantly enlarged cardiacsilhouette (water-bottle sign) and bilateral pleural effusion. This corresponds to the chest radiograph

Initial diagnostic workup included a 12-lead electrocardiogram (ECG) demonstrating sinus rhythm (70 bpm) with generalized low QRS voltage across all leads, non-specific repolarization abnormalities, and electrical alternans in leads V2-V3, findings highly suggestive of a large pericardial effusion. Additionally, chest X-ray revealed bilateral pleural effusion and a significantly enlarged cardiac silhouette (Fig. 1).

**Fig. 2:**
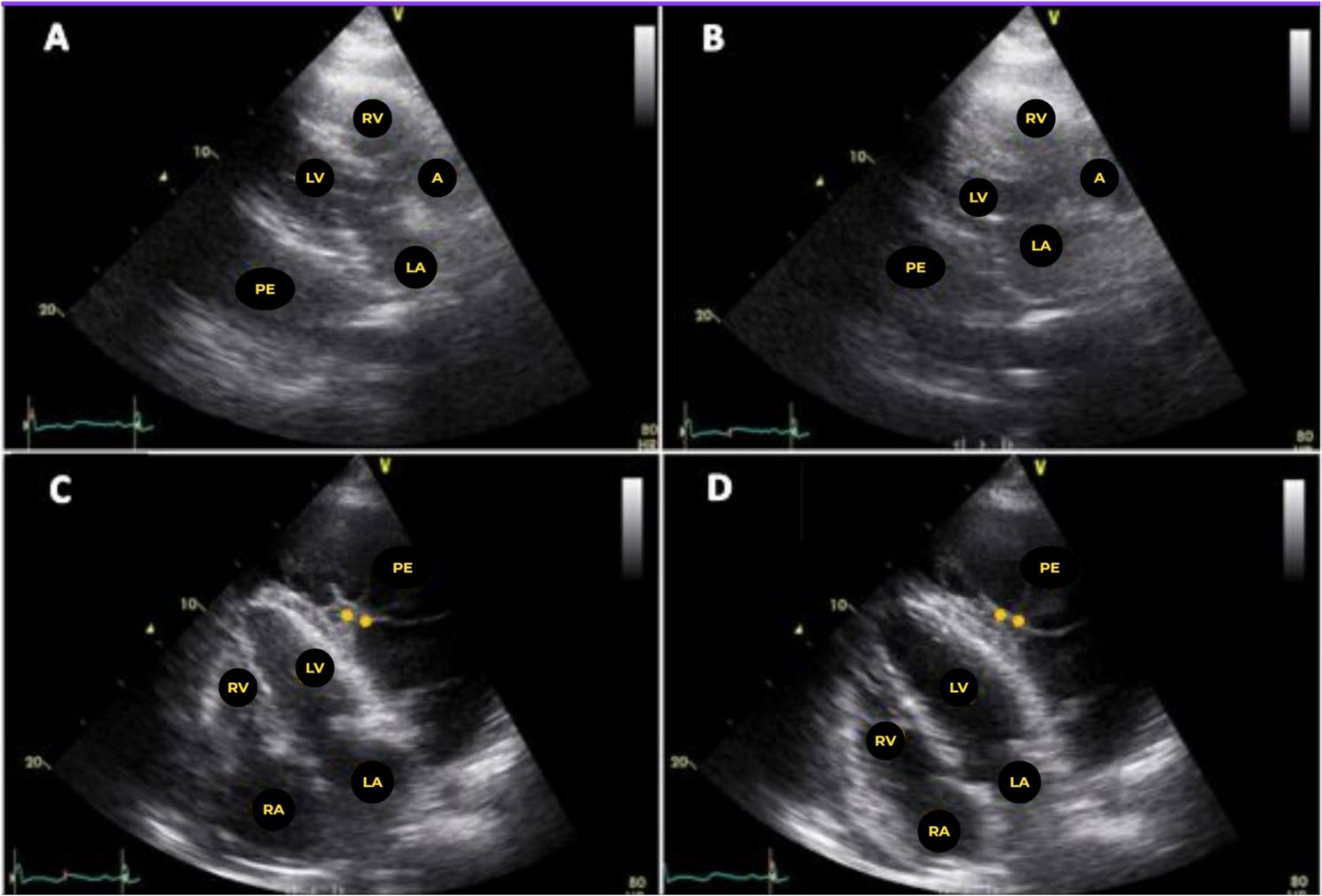
Transthoracic echocardiogram. **A** and **B**: Parasternal long-axis views demonstrating extensive circumferential pericardial effusion (DP) without evident diastolic compression of the right ventricular free wall. **C**: Four-chamber apical view in systole showing the magnitude of the pericardial effusion (DP). **D**: Four-chamber apical view in diastole highlighting the presence of fibrin strands (Fibrin) within the effusion, suggesting an exudative or malignant process. RV = Right Ventricle, LV = Left Ventricle, RA = Right Atrium, LA = Left Atrium, PE = Pericardial Effusion A = Aorta **Fibrin strands

Transthoracic echocardiography (TTE) revealed a limited acoustic window. Despite this limitation, a severe circumferential pericardial effusion was identified, characterized by numerous fibrin strands without overt signs of cardiac tamponade (e.g., early diastolic collapse of the right ventricle). Biventricular systolic function was preserved, and no valvular abnormalities were noted (Fig. 2).

Additionally, the patient experienced self-limited KDIGO 1 acute kidney injury. A comprehensive laboratory evaluation was performed. NT-proBNP levels were markedly elevated (1220pg/mL), consistent with acute decompensated heart failure. Cardiac biomarkers, including high-sensitivity troponin I, were within normal limits. Acute kidney injury was classified as KDIGO stage 2, predominantly ischemic in nature. Liver function tests were within normal ranges, with mild hypoalbuminemia (3.1 g/dL). Autoimmune, infectious, and endocrine causes of polyserositis were systematically excluded.

Given the rapid progression of symptoms, absence of etiology and acute kidney injury limiting contrast studies, it was decided to transfer to the intensive care unit for hemodynamic monitoring. The heart team evaluated the case and an assessment of survival and prognosis was undertaken prior to performing invasive studies such as pericardiocentesis.

**Fig. 3:**
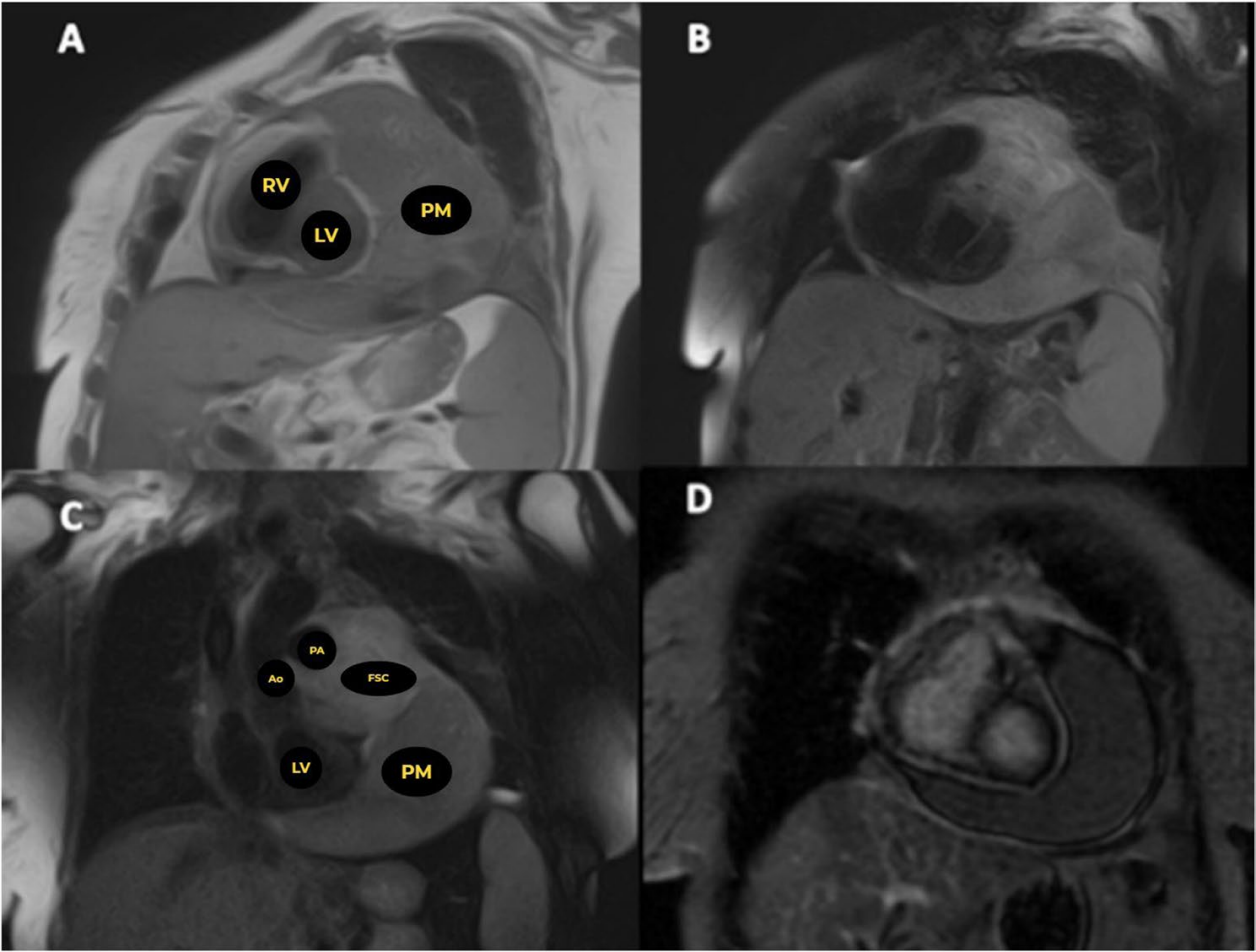
Cardiac Magnetic Resonance Imaging. **A** and **B**: T1 and T2-weighted sequences showing a voluminous, circumferential, and heterogeneous Pericardial mass (PM) associated with a large posterior hemopericardium. **C**: Short-axis T2-weighted sequence with fat suppression (STIR) revealing a heterogeneous mass with a superior Fatty component (FSC) partially encasing the Pulmonary artery (PA) and the Ascending aorta (Ao). **D**: T1-weighted sequence with Late gadolinium enhancement (LGE) demonstrating intense peripheral enhancement followed by progressive heterogeneous central enhancement, supporting the diagnosis of a malignant mesenchymal tumor. RV = Right ventricle, LV = Left ventricle, PM = Pericardial mass, Ao = Aorta, PA = Pulmonary artery PM = pericardial mass FSC = Fatty solid component of pericardial mass

After resolution of the acute kidney injury and given clinical stability, further investigation with contrast-enhanced CT of the neck, chest, and abdomen showed a significant pericardial hypodensity (up to 64 mm thick) with a solid posteromedial component and heterogeneous enhancement, exerting a compressive effect on the left ventricular lateral wall and the pulmonary trunk. Extensive lymphadenopathy was identified in the neck, anterior and middle mediastinum (with central necrosis), and mesentery, alongside moderate ascites (the latter evidenced in ECO TT) with compression of the lateral wall of the left ventricle; sagittal projection showed circumferential involvement, a key finding to determine a heterogeneous mass of pericardial origin. Cardiac magnetic resonance (CMR) identified a large anterior mediastinal mass (92 × 88 × 75 mm), primarily localized in the left paramedian region. The mass exhibited heterogeneous signal intensity, with T2-weighted fat-suppressed (FS) and STIR sequences showing hyperintensity in the superior portion, while T1-weighted sequences remained isointense relative to the myocardium. The lesion encased the distal ascending aorta without luminal compromise and enveloped the main pulmonary artery, with SSFP cine sequences demonstrating adherence and narrowing at the bifurcation. Findings of reduced biventricular volumes, a flattened interventricular septum, and paradoxical septal motion were highly suggestive of constrictive physiology (Fig. 3).

**Fig. 4:**
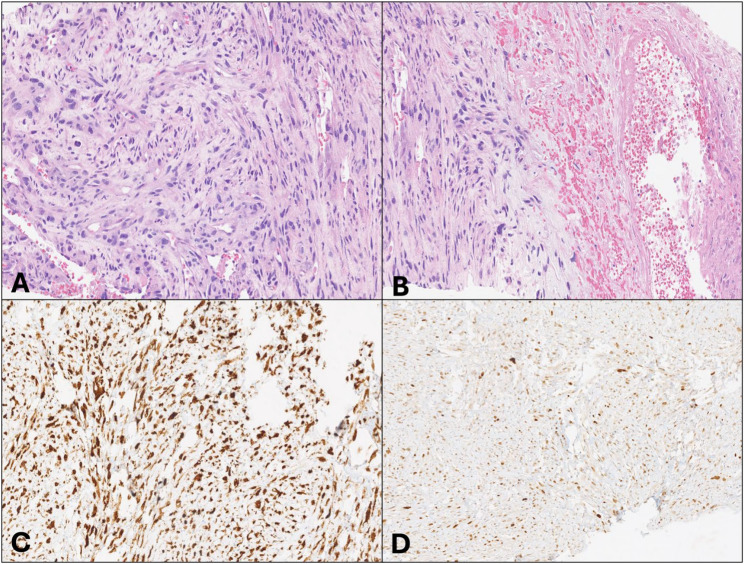
Histopathological analysis: **A** Dedifferentiated liposarcoma (H&E): high-grade malignant mesenchymal neoplasm composed of pleomorphic spindle cells with marked nuclear atypia and frequent mitotic activity. **B** Dedifferentiated liposarcoma (H&E): high-grade pleomorphic sarcoma with abrupt transitions between viable tumor and extensive areas of geographic necrosis. **C** CDK4 immunohistochemistry: strong nuclear positivity in tumor cells, supporting the diagnosis of dedifferentiated liposarcoma. **D** MDM2 immunohistochemistry: diffuse nuclear positivity in tumor cells, supporting the diagnosis of dedifferentiated liposarcoma

Concurrently mediastinal and low cervical lymphadenopathies were also identified, leading to an initial diagnostic impression of lymphoma or cardiac primary tumor (less probably). An ultrasound-guided percutaneous core needle biopsy (Tru-cut) of the solid component of the anterior mediastinal mass was performed using an 18G needle. The histopathological analysis revealed a high-grade pleomorphic spindle cell sarcoma. Immunohistochemistry results showed strong and diffuse nuclear positivity for MDM2 and CDK4, while other markers (including Desmin, SMA, CK AE1/AE3, CD34, S100, and STAT6) were negative, confirming the diagnosis of a dedifferentiated liposarcoma (Fig. 4).

The patient was transferred to an oncology referral center, where a palliative chemotherapy plan was initiated using the MAI regimen (Mesna, Adriamycin [Doxorubicin], and Ifosfamide). A thoracic surgery consultation was requested to evaluate palliative debulking for symptom relief; however, on the second day of the first treatment cycle, the patient’s clinical status deteriorated rapidly. This decline was characterized by worsening dyspnea, increased peripheral edema, and new-onset acute kidney injury (serum creatinine 1.5 mg/dL). The clinical course progressed to hypoxemic respiratory failure and obstructive shock. In the context of advanced malignant disease under palliative management, and given the previously documented extensive cardiac and mediastinal involvement, the terminal event was clinically consistent with acute hemodynamic decompensation, most likely driven by cardiac tamponade and concomitant pulmonary edema.The patient died shortly thereafter following a rapidly progressive clinical course.

**Fig. 5:**
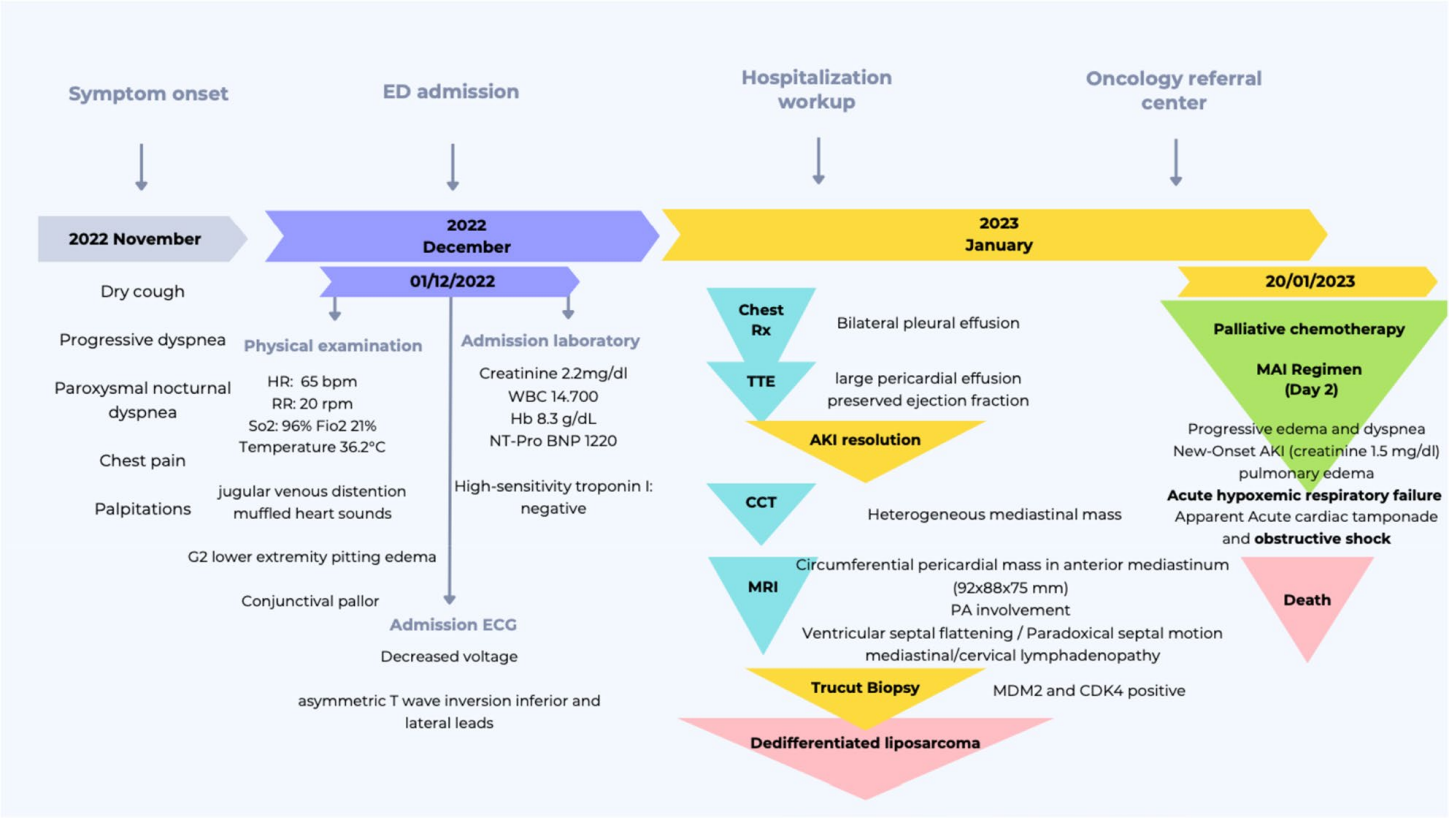
Clinical timeline summarizing key diagnostic and therapeutic milestones

To further illustrate the clinical course and decision-making milestones, we have added a diagnostic and management timeline (Fig. 5), summarizing the progression from symptom onset to the final outcome.

## Discussion

Primary cardiac tumors are rare, with an incidence of less than 0.3% of all tumors [4]. Pericardial masses, specifically, are categorized into non-neoplastic, primary benign, and malignant lesions. Non-neoplastic etiologies include congenital cysts or diverticula, as well as reactive or inflammatory processes such as rheumatoid nodules and infectious lesions (e.g., echinococcal or tuberculous cysts). Primary benign neoplasms are infrequent, consisting mainly of lipomas, lipoblastomas, and teratomas. In contrast, malignant neoplasms are predominantly secondary; metastatic involvement is reported to be 100 to 1,000 times more frequent than primary malignant tumors, occurring in up to 14.2% of patients with documented metastatic disease. These cases frequently manifest as hemorrhagic effusions and are primarily of epithelial origin, with lung and breast carcinomas being the most commonly described sources.

Primary malignant pericardial lesions are exceptionally rare and include primary pericardial mesothelioma—the most frequent type, occasionally associated with prior radiation therapy—and various sarcomas. While angiosarcomas are the most common primary malignancy of the heart, other histological subtypes such as synovial sarcomas and liposarcomas have been reported. Primary pericardial lymphomas, predominantly diffuse large B-cell types, and malignant germ cell tumors also constitute part of the differential diagnosis [5] Among these, primary pericardial liposarcoma remains a clinical challenge due to its infrequent occurrence and the necessity of distinguishing it from other mesenchymal tumors through advanced molecular and imaging techniques.

There are at least four subtypes of liposarcoma described: well-differentiated, dedifferentiated, myxoid, and pleomorphic. The pleomorphic subtype is the 8 and is generally associated with a poor prognosis, particularly in advanced or unresectable cases. In our case, the identification of MDM2 and CDK4 amplification was crucial. Recent evidence suggests that MDM2 amplification is often more consistent and quantitatively greater than CDK4 amplification, supporting its central role in liposarcoma tumorigenesis. Although MDM2/CDK4 co-amplification has been associated with poorer clinical outcomes, it may not represent an independent prognostic factor, likely reflecting the influence of additional clinicopathological variables such as surgical margins and tumor size [6]. 

Reported long-term survival (approximately 8.3 years) has been described primarily in highly selected patients who underwent complete surgical resection (R0), a scenario that was not applicable in our case. However, other studies have shown a 12-month survival, and the 5-year follow-up post-tumor recurrence after resection has been reported at 40% [7–9]. Symptoms are typically nonspecific; in previously reported cases, cough and dyspnea occurred in 74% of patients, while chest pain and palpitations were seen in 40% [10–12].

Multimodal imaging plays a central role in the study of cardiac masses (CM), including malignant primary cardiac tumors. Although histopathological tests are the gold standard for their study and diagnosis, imaging findings can often not only locate the lesion but also predict with high probability whether it is a benign or malignant process, and help narrow the differential diagnosis [3]. Key points to consider include pleural effusion and an increased cardiac silhouette on chest X-ray. Echocardiography is typically the first-line study for suspected cardiac masses, aiming to define characteristics such as motility, size, and location. In our case, the morphological characteristics of the mass combined with the poor acoustic window allowed us only to observe the liquid component of the mass through a 4-chamber apical view. Cardiovascular magnetic resonance imaging (CMR) is considered the next study after echocardiography due to its excellent multiplanar resolution and the ability to differentiate signals from various tissue types. The use of cardiac CT and MRI allowed us to establish the pericardial origin of the mass, as well as its heterogeneous component, including a fatty component in the upper part. The above allowed us to expand the range of differential diagnoses and guide the site for taking a biopsy.

There are no clinical practice guidelines or high-quality evidence for the management of cardiac undifferentiated liposarcoma. Recent literature reviews suggest that any undifferentiated sarcoma in this location should undergo complete resection or debulking for symptomatic improvement. While surgical resection is the preferred treatment if feasible, different therapeutic regimens have been proposed in case reports, including halichondrin analogs such as eribulin. In our case, the morphology and size of the lesion, with circumferential pericardial involvement and extension to mediastinal structures (specifically the left pulmonary artery), were considered clear limitations for excisional treatment. Nevertheless, palliative debulking was intended; however, the aggressive course of the disease, resulting in death on the second day of the first chemotherapy cycle, rendered it infeasible.

Complications, like other cardiac tumors, may include peripheral embolization in 85%, left atrial involvement in 80%, less frequently in the right atrium, arrhythmias, and cardiac tamponade [13], the latter, leading to the death of our patient.

In our case, several factors likely contributed to the fatal outcome. Progressive peripheral edema and acute kidney injury, together with worsening dyspnea preceding the initiation of the MAI regimen, suggested a state of underlying cardiac dysfunction with associated pulmonary congestion. This baseline hemodynamic vulnerability, compounded by the patient’s frailty and the initiation of cytotoxic therapy—including Adriamycin—may have further destabilized the clinical condition, ultimately leading to hypoxemic respiratory failure and obstructive shock.

The presence of an obstructive hemodynamic phenotype is supported by the known compromise of the left pulmonary artery and previously described septal flattening with paradoxical motion, findings that are consistent with acute cardiac tamponade as the most plausible terminal mechanism. Although tumor lysis syndrome was considered in the differential diagnosis, it was subsequently excluded based on clinical and laboratory data. Given that the patient was under a palliative care plan, escalation to advanced resuscitative measures or additional confirmatory diagnostic studies was not pursued, as these were considered non-beneficial.

## Conclusion

This case highlights the diagnostic complexity of rare primary cardiac malignancies, particularly pericardial liposarcoma, which may initially mimic large pericardial effusion. The integration of clinical data with multimodal imaging including echocardiography, CT, and cardiac MRI was essential for identifying the mass and guiding tissue diagnosis. Given the aggressive behavior, lack of standardized treatment protocols, and poor prognosis in unresectable cases, early recognition through imaging may facilitate more timely interventions. Further research is needed to better define imaging criteria and optimal management strategies for cardiac sarcomas.

## Data Availability

Not applicable.

## Abbreviations

PCT: Primary cardiac tumors
ED: Emergency department
ECG: Electrocardiogram
ECOTT: Transthoracic echocardiogram
CTA: Computed tomography angiogram
CM: Cardiac mass
CMR: Cardiovascular magnetic resonance
